# Simulation and Evaluation of Spring Maize Growth Under Drip Irrigation with Fully Biodegradable Film Mulching Based on the DSSAT Model

**DOI:** 10.3390/plants13213027

**Published:** 2024-10-29

**Authors:** Yanhui Jia, Haibin Shi, Qingfeng Miao, Xiulu Sun, Yayang Feng

**Affiliations:** 1 Shandong Facility Horticulture Bioengineering Research Center, Weifang University of Science and Technology, Weifang 262700, China; jyh_5151@126.com; 2 College of Water Conservancy and Civil Engineering, Inner Mongolia Agricultural University, Hohhot 010018, China; imaumqf@imau.edu.cn; 3 Autonomous Region Collaborative Innovation Center for Integrated Management of Water Resources and Water Environment in the Inner Mongolia Reaches of the Yellow River, Hohhot 010018, China; 4 Farmland Irrigation Research Institute, Chinese Academy of Agricultural Sciences (CAAS), Xinxiang 453002, China; sunxiulu@caas.cn

**Keywords:** fully biodegradable mulch film, subsurface drip irrigation, DSSAT model, yield, water use efficiency

## Abstract

Fully biodegradable mulch film enhances temperature and moisture retention during the early stages of maize growth while naturally degrading in the later stages, providing an environmentally friendly alternative to conventional plastic mulch films. However, there is no consensus on its impact on maize growth and yield. The present study utilized field test data from spring maize covered with fully biodegradable mulch film in the Xiliaohe Plain, aiming to improve the Decision Support System for Agrotechnology Transfer (DSSAT) model while focusing on soil temperature, irrigation, rainfall, and evapotranspiration. The parameters of the DSSAT model were calibrated and validated using field test data from 2016 to 2018. The improved DSSAT model accurately simulated the maize growth process under various induction periods of fully biodegradable mulch film. The simulation accuracy of this model was as follows: MRE < 10%, nRMSE < 12%, and *R*^2^ ≥ 0.80. Moreover, the yield of spring corn covered with fully biodegradable mulch film was predicted using meteorological data from 2019 to 2023. This study suggests that regions such as the Xiliaohe Plain, which share climatic conditions, should opt for fully biodegradable mulch film with an induction period of approximately 80 days to ensure high yields across different hydrological years.

## 1. Introduction

The Xiliao River Plain is located in the Golden Belt of maize production and is an important grain-producing area in China [1]. However, spring maize in this region is highly susceptible to cold damage and suffers from inadequate water resources [2]. The Xiliao River has experienced years of reduced flow, making groundwater extraction the primary irrigation method. This has resulted in the formation of significant groundwater cones in the depression, severely constraining the regional production capacity [3]. Subsurface drip irrigation technology effectively alleviates issues related to drought and cold conditions affecting spring maize in the Xiliao River Plain [4]. However, the long-term use of conventional plastic mulch film leads to “white pollution” [5]. Additionally, rainfall in the Xiliao River Plain is unevenly distributed throughout the year, with over 60% of the annual rainfall occurring from July to September. The use of mulch can hinder rainwater utilization [6,7]. In this context, promoting fully biodegradable mulch film that provides the same warming and moisture retention effects as those of conventional plastic mulches before degradation [8] while also increasing rainwater utilization during the maize growth phase is crucial. These biodegradable films naturally decompose into CO_2_, H_2_O, and organic matter in the later growth stages [9], representing an important measure for ensuring the green and sustainable development of subsurface drip irrigation technology in the Xiliao River Plain and safeguarding national food security [10].

Recent research on fully biodegradable mulch film has primarily focused on the induction phase, warming, and moisture retention effects and their impacts on maize yield and water use efficiency (WUE). Chen et al. [11] showed that in the Hetao Plain, after the degradation of fully biodegradable mulch film, soil surface evaporation increases, leading to a reduced water retention capacity for maize in the mid-to-late growth stages. Transpiration under fully biodegradable mulch was 15.6% higher than that under conventional polyethylene (PM) treatment, whereas the yield decreased by 9.9% relative to that for PM. A study of potatoes has shown that evapotranspiration (ET) and yield do not differ significantly between fully biodegradable and conventional mulch, and the limited impact of fully biodegradable mulch on ET could be explained by the high canopy coverage of potatoes [12]. In a study of maize, Saglam et al. [7] found no significant difference in soil moisture between fully biodegradable mulch and PM treatment; after rainfall, soil moisture in areas covered by fully biodegradable mulch was even higher than that in PM-covered areas. Three-year maize trials in the Weinan Plain have demonstrated that the average yield and WUE are significantly higher for moderately degraded fully biodegradable mulch than for conventional mulch and both slowly and rapidly degrading biodegradable film [13]. Feng et al. [10] studied spring maize in eastern Inner Mongolia, revealing that in wet and average water years, fully biodegradable mulch could achieve higher yields; however, yields did not differ significantly from those for the PM treatment. However, during dry years, the yield and WUE were significantly lower for the fully biodegradable mulch than for the PM treatment. These findings highlight the potential for variation in climatic conditions, hydrological year types, and maize types across regions to lead to differences in water consumption, maize yield, and WUE under fully biodegradable mulch coverage. However, field experiments have not thoroughly investigated the effects of these parameters under fully biodegradable mulch use, and such experiments are time-consuming, labor-intensive, and costly. Combining field experiments with modeling approaches can improve the estimation of maize yields.

The DSSAT (decision support system for agrotechnology transfer) model is widely used to predict maize yields owing to its user-friendly interface and high simulation accuracy. In the DSSAT model, ET and maize yield are primarily estimated based on soil temperature, rainfall, and solar radiation. However, this model does not include a mulch cover module and is mainly used to simulate uncovered field plots. Shen et al. [14] utilized an improved MDSSAT model to simulate maize yields under PM mulch, considering the impact of damaged areas caused by corn stover growth holes on soil evaporation. However, the effect of these damaged areas on soil temperature was not considered. Chen et al. [15] proposed a BDSSAT model to simulate the effects of a fully biodegradable mulch cover by considering the mulch degradation rate and its impact on soil temperature and maize yield. This model aimed to predict maize yield changes in Inner Mongolia under future climate change scenarios. Overall, these studies indicate that the DSSAT model can effectively simulate maize growth under film mulching conditions and accurately predict maize yield and water productivity when using fully biodegradable mulch film. However, the reliability of these simulations is highly contingent upon the careful selection of maize genetic parameters, field management practices, and soil characteristics. Therefore, it is crucial to enhance the DSSAT model using field test data, optimize both maize genetic and input parameters, and validate the model to accurately predict maize yield and water productivity under fully biodegradable mulch film. This approach will ultimately facilitate the optimization of the induction period for this film. 

The objectives of this study were as follows: (1) improving the DSSAT model to accommodate fully biodegradable mulch conditions, considering the impact of different stages of mulch coverage on rainfall utilization; (2) considering the one-dimensional point-based nature of the DSSAT model and localized drip irrigation system, necessitating adjustments to the irrigation amounts to enhance the accuracy of soil moisture simulations near the drip tape; (3) optimizing genetic parameters for spring maize using field observation data from 2016 to 2018 to calibrate and validate the DSSAT model under fully biodegradable mulch coverage; and (4) utilizing meteorological data from 2019 to 2023 to simulate and predict spring maize yield under different induction periods with fully biodegradable mulch coverage. Based on these simulations, an optimal induction period for biodegradable mulch in the Xiliaohe Plain and similar climatic regions was proposed. This study offers a scientific basis and technical support for predicting maize productivity under fully biodegradable mulch in the semi-arid regions of Northeast China, particularly in the context of climate change and the efficient management of agricultural water and heat resources. 

## 2. Results

### 2.1. Fully Biodegradable Mulch Film Damage Ratio

**Figure 1:**
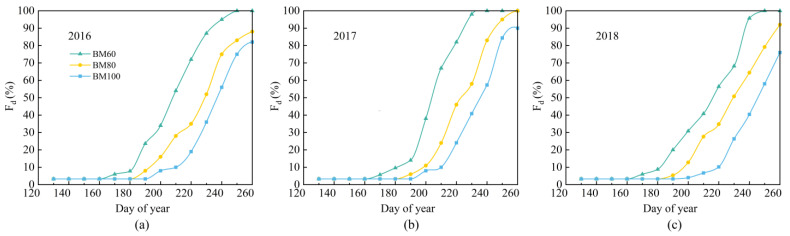
Damage ratios of fully biodegradable film at different induction periods in 2016 (**a**), 2017 (**b**), and 2018 (**c**).

In this study, a seed drill was used to create corn-planting holes with a radius of approximately 4 cm. The fully biodegradable film gradually degraded until the induction period. The coverage based on actual measurements in field trials is illustrated in Figure 1. Prior to the induction period, all three types of fully biodegradable film exhibited high degrees of integrity. However, 30 d after the induction period, the damage ratio for all three types exceeded 40%. Subsequently, the moisture retention effect provided by the biodegradable film was largely lost, and the obstruction effects of the film on rainfall were negligible.

### 2.2. Temperature Increase Compensation Coefficient

**Figure 2:**
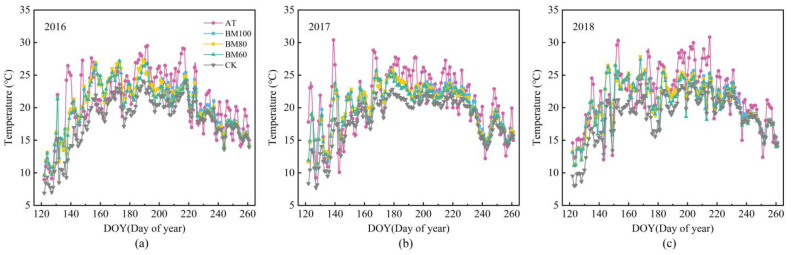
Daily changes in daily average temperature and soil temperature at a depth of 5 cm under different mulching treatments in 2016 (**a**), 2017 (**b**), and 2018 (**c**).

The dynamic changes in the daily average soil temperature at a depth of 5 cm for the fully biodegradable film-covered treatments and non-covered controls during 2016, 2017, and 2018 are illustrated in Figure 2. From the time of sowing until the corn reached the tasseling stage (approximately 70 days after sowing), the fully biodegradable film coverage exhibited a significant warming effect, with an average soil temperature increase of 2.65 °C compared with that for the non-covered treatment. As spring corn entered the silking stage (about 80 days after sowing for the fully biodegradable film-covered treatment and about 90 days for the non-covered treatment), the canopy coverage of the corn reached its maximum value. Subsequently, the warming effects for both covered and uncovered treatments decreased gradually. Additionally, the warming effect of the biodegradable film in the BM60 and BM80 treatments decreased over time owing to damage and degradation, leading to a gradual loss of the warming effect. The warming effect of the BM100 treatment also began to diminish at 110 d post-sowing.

**Figure 3:**
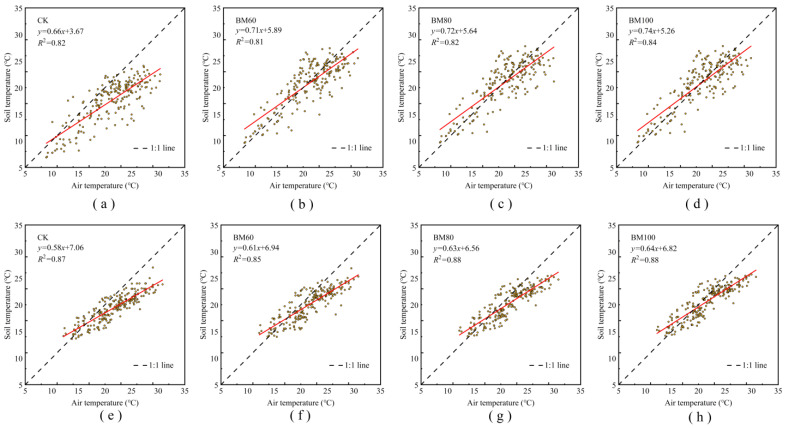
Relationship between the daily average air temperature and soil temperature at a depth of 5 cm under different mulch treatments at 0–70 d (**a**–**d**) and 71–140 d (**e**–**h**).

Soil temperature is primarily influenced by the air temperature. We set the daily average air temperature as the independent variable and analyzed its linear correlation with the daily average soil temperature at a depth of 5 cm using a 70-day post-sowing period as the threshold (Figure 3). The regression slopes for BM60, BM80, and BM100 from 0 to 70 d post-sowing were 0.71, 0.72, and 0.74 (Figure 3b–d), which were all higher than that of the non-mulched control treatment (CK) at 0.66 (Figure 3a). These results indicate that the use of fully biodegradable mulch significantly enhances warming compared with that for the non-mulched treatment. From 70 to 140 d after sowing, the regression slopes for BM60, BM80, BM100, and CK were 0.61, 0.63, 0.64, and 0.58 (Figure 3e–h). The gap between the CK and mulched treatments decreased, suggesting that the warming effect of the mulch treatments gradually weakened over time. The longer the duration of fully biodegradable mulch coverage, the greater the slope of the regression equation, further confirming the warming effect of mulch. As shown in Figure 3, the correlation coefficients (*R*^2^) of the linear equations for the daily average air temperature and daily average soil temperature at a depth of 5 cm for all treatments were greater than 0.8, indicating a strong correlation and high precision. Therefore, using the linear equations shown in Figure 3 and the daily average air temperatures from 2019 to 2023, we calculated the soil temperature at a depth of 5 cm for different fully biodegradable mulch treatments.

Based on the linear relationship between the soil temperature and air temperature in the 5 cm soil layer for fully biodegradable mulch treatments and non-mulched treatments, we utilized air temperature data for 2019 to 2023 to estimate the corresponding soil temperatures. The Temperature Increase Compensation Coefficient was calculated using Equations (8) and (9) (Table 1). The compensation coefficients for the fully biodegradable mulch treatments BM60, BM80, and BM100 during the sowing to emergence stage were 1.50. During the emergence to elongation stage, differences in compensation coefficients began to emerge, with average values of 0.36, 0.40, and 0.42 for BM60, BM80, and BM100, respectively. In the elongation to tasseling stage, the warming effect of the mulch decreased gradually, with average values of 0.11, 0.14, and 0.18 for BM60, BM80, and BM100, respectively. As the growing season progressed, the compensatory effect of air temperature on soil temperature gradually weakened, and the coefficient of variation (CV) increased. This indicates that the coverage ratio is an important factor influencing the soil temperature.

### 2.3. Calibration of the DSSAT Model Under Fully Biodegradable Mulch

Using the improved model, we calibrated various parameters for maize varieties based on field trial data from 2016, specifically for the fully biodegradable mulch treatment, BM60. These parameters included the phenological stage, LAI, yield, and soil moisture content. First, the Generalized Likelihood Uncertainty Estimation (GLUE) parameter adjustment method for DSSAT was used. Parameters were further refined by manually comparing the simulated and observed values. The genetic coefficients used in this study are listed in Table 2. The CERES-maize model incorporates six genetic parameters for maize. Four of these parameters were related to maize growth and development (P1, P2, P5, and PHINT), and two parameters influenced maize yield (G2 and G3).

**Figure 4:**
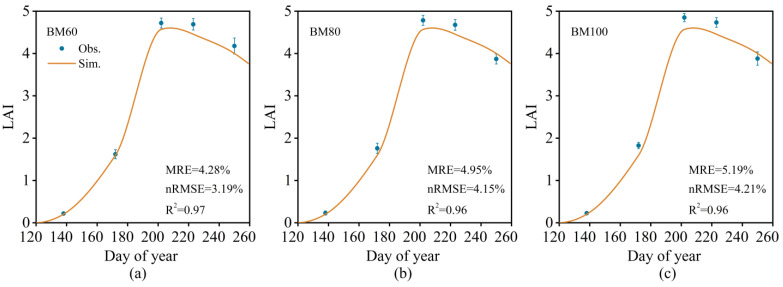
Measured values of LAI in 2016BM60 (**a**), 2016BM80 (**b**), and 2016BM100 (**c**) and simulated values based on the improved model.

**Figure 5:**
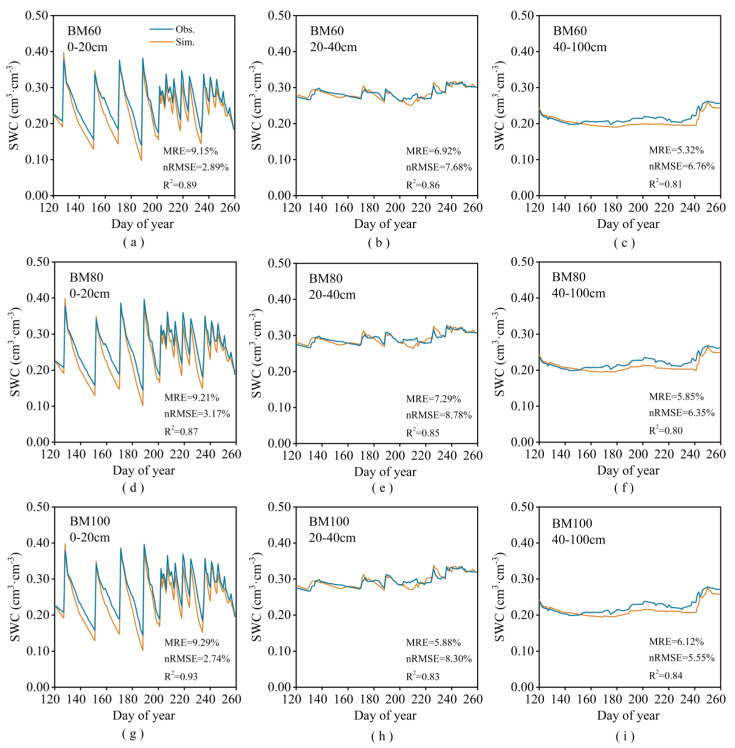
Measured soil water contents in the 0–20 cm (**a**,**d**,**g**), 20–40 cm (**b**,**e**,**h**), and 4–100 cm (**c**,**f**,**i**). Soil layer and simulated values using the improved DSSAT model for BM60, BM80, and BM100 in 2016.

The improved model accurately simulated the LAI and soil moisture content for the fully biodegradable mulch treatment (BM60), as shown in Figure 4 and Figure 5. Additionally, it effectively modeled the phenological stage, yield, and ET, as presented in Table 3. The calibrated model demonstrated a high degree of fit for the number of growing days, yield, and ET, with a mean relative error (MRE) for the simulated and observed values remaining within 5%. The model also showed good performance for the soil moisture content and LAI, with the MRE and normalized root mean square error (nRMSE) under 10% and *R*^2^ values exceeding 80%. This indicates that the improved model can accurately simulate soil water movement and spring maize growth under biodegradable mulch. As shown in Figure 5, the soil water content at depths of 0–40 cm varied significantly. Under drip irrigation, maize roots were primarily distributed within this depth range, which is crucial for water uptake. Additionally, irrigation and rainfall primarily affected the moisture content in the top 0–20 cm of the soil layer, resulting in minimal impact on the moisture levels in the soil below 40 cm.

### 2.4. Model Validation Under Fully Biodegradable Mulch

**Figure 6:**
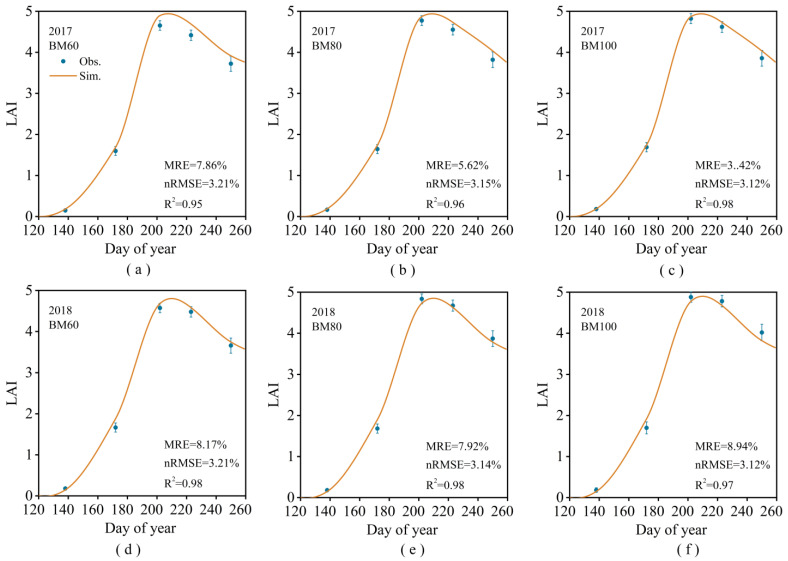
Measured values of LAI and simulated values using the improved DSSAT model for 2017BM60 (**a**), 2017BM80 (**b**), 2017BM100 (**c**), 2018BM60 (**d**), 2018BM80 (**e**), and 2018BM100 (**f**).

**Figure 7:**
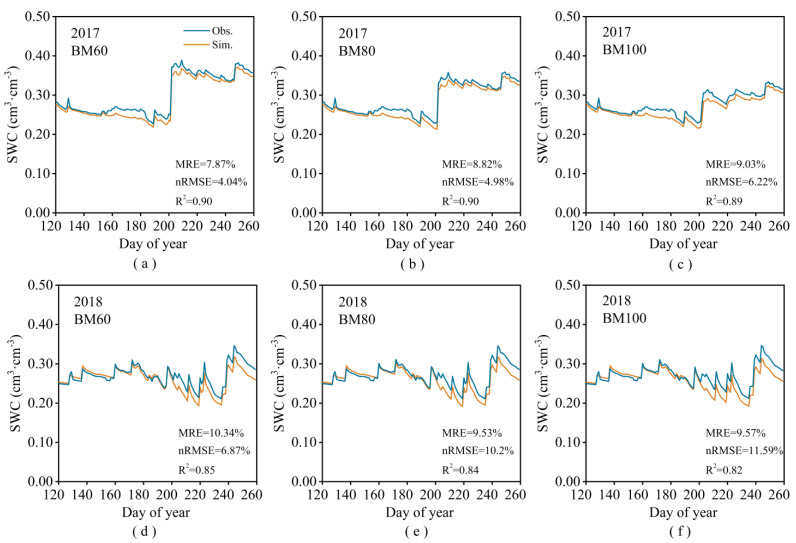
Measured values of average soil moisture in the 0–1 m soil layer and simulated values based on the improved DSSAT model for 2017BM60 (**a**), 2017BM80 (**b**), 2017BM100 (**c**), 2018BM60 (**d**), 2018BM80 (**e**), and 2018BM100 (**f**).

The accuracy of the calibrated model was validated using observational data from experiments conducted in 2017 and 2018. The relevant statistical indicators for the phenological stages, yields, and ET are presented in Table 3. The measured and simulated values of the LAI were very similar (Figure 6). The correlation between the simulated and observed values for the soil moisture content was slightly lower than that for the LAI (Figure 7). This indicates that the improved model demonstrates high simulation accuracy for the LAI, phenological stages, yield, soil moisture content, and ET of spring corn. The coefficients of determination (*R*^2^) for the relationship between the simulated and observed values were greater than 80%, and the mean relative errors (MRE) were less than 10%.

### 2.5. Yield Prediction Under Fully Biodegradable Mulch

To further evaluate the impacts of different induction periods of fully biodegradable mulch on the yield and WUE of spring maize, the DSSAT model was employed along with meteorological data from 2019 to 2023. Maize yields under various induction periods of biodegradable mulch were predicted. Based on the recommended irrigation volume for maize drip irrigation in the Xiliao River Plain region, as well as rainfall data for the study area, the model utilized irrigation volumes of 183 mm for normal water years and 105 mm for wet years. The model inputs for the different induction periods, including rainfall, yield, and WUE, are summarized in Table 4. From 2019 to 2020, classified as normal water years, the simulated yield ranged from 12,140.05 to 13,692.62 kg·ha^−1^, with WUE values between 26.94 and 29.85 kg·mm^−1^·ha^−1^, showing no significant difference compared with the measured yield of 12,109.01 to 13,301.33 kg·ha^−1^ in 2016. In 2023, which was classified as a wet year, the yield was substantially lower than that in normal years. This decline can be attributed to the rainfall in July (291 mm), which accounted for 70% of the precipitation during the growing season. Excess rainfall can lead to overly wet soil conditions, which negatively affect root respiration and nutrient absorption. Additionally, continuous heavy rainfall during the tasseling stage affects maize pollination, further affecting yield. Based on the monitored yield data for 2016 to 2018 and the model predictions for 2019 to 2023, in the Xiliao River Plain and similar climatic regions, a biodegradable mulch with an induction period of approximately 80 days is recommended to ensure high yields across various hydrological conditions.

## 3. Discussion

Soil temperature is a key factor determining maize growth and is influenced by various factors, such as solar radiation, soil cover, air temperature, irrigation, and rainfall. The warming effect of mulch shows substantial spatiotemporal variation. Chen et al. [16] first reported that mulching increased the accumulated soil temperature in the plowed layer during the early growth stages of cotton, compensating for insufficient air temperature. The warming effect of mulch on the plowed layer was significant, although it diminished after July. Wang et al. [17] indicated that plastic film coverage created favorable conditions for maize emergence and early growth, resulting in an average yield increase of 4.9% over the entire growth period compared with that for bare soil and significantly increasing soil temperatures. Air temperature directly affected soil temperature at a depth of 5 cm under mulched conditions, demonstrating a linear positive correlation. This relationship has been validated in field experiments using various maize, such as maize, wheat, and cotton [14,18,19]. Extensive research has shown that fully biodegradable film exhibits the same warming effect as that of conventional plastic film before degradation [20,21]. Yang et al. [18] showed that in mulched maize, the most pronounced effects occurred just before the maximum canopy cover (prior to tasseling), providing a compensatory coefficient for air temperature in mulched maize in western Liaoning. Understanding the relationship between soil temperature under mulch and air temperature, as well as quantifying the compensatory effect, is crucial for converting air temperature data into soil temperature calculations for mulched maize. Similarly, this study utilized a linear equation for air temperature and the 5 cm soil temperature alongside temperature data for 2019 to 2023 to predict the air temperature compensation coefficients for different growth stages of maize under various induction periods of a fully biodegradable film. The soil temperature under fully biodegradable film was influenced by the rate of film degradation; once the film deteriorated, the warming effect decreased gradually.

Soil moisture is another critical factor affecting maize yield and is primarily influenced by irrigation, rainfall, soil cover, and maize growth. During the seedling stage, maize requires relatively low water, with peak demand occurring from the jointing to grain-filling stages. Water requirements decrease significantly after grain filling [22]. Therefore, in the Xiliao River Plain, where rainfall is concentrated in July and August, it is essential for fully biodegradable film to remain intact during the early growth stages to reduce soil evaporation, whereas post-degradation, they should enhance rainwater utilization efficiency [23]. Li et al. [24] modeled mulched cotton in Xinjiang, which receives minimal rainfall; however, in eastern Xinjiang, once rainfall reaches moderate levels, it can move laterally to areas covered by mulch. Consequently, this study improved the rainfall input data for the model. Fu et al. [25] pointed out that because drip irrigation is a localized irrigation method, using actual irrigation amounts in the model may reduce the soil moisture content near the drip irrigation zone. Therefore, the actual amount of irrigation was adjusted based on the depth of the soil moisture layer. This study also improved the water balance equations for the soil moisture content before and after irrigation. The newly developed model accurately simulates soil moisture dynamics by refining the input values for rainfall and irrigation.

Because the management module of the DSSAT model does not account for soil cover, this study adopted the method developed by Shen et al. [14] to incorporate evaporation ratio coefficients. After the modifications, the model was calibrated using data from 2016 for spring maize genetic parameters and validated against experimental data from 2017 and 2018. The model showed high simulation accuracies for the LAI, phenology, yield, soil moisture, and ET of spring maize, with correlation coefficients (*R*^2^) exceeding 80% and an MRE below 10%. During the prediction phase, the model yield forecasts were closely aligned with the observed experimental yields. Thus, the calibrated post-improvement model can effectively simulate the growth and development of spring maize under fully biodegradable film coverage, providing a reliable analytical tool for assessing water consumption and field moisture management in maize grown under such conditions.

## 4. Materials and Methods

### 4.1. Field Experiment

A spring maize drip irrigation experiment under fully biodegradable mulch coverage was conducted in the core experimental area with subsurface drip irrigation in Kezuozhongqi, Tongliao City, Inner Mongolia [44°6′ N, 122°21′ E, 173.5 m above sea level (m.a.s.l.)]. The average annual rainfall in the study area from 1951 to 2018 was 324 mm, with 80% of the rainfall concentrated during the spring maize growth period (May to September). The long-term average temperature was 20.2 °C, and the average annual sunshine duration was 3254.1 h. The groundwater depth in the experimental area was approximately 8.5 m, and the soil texture and hydraulic parameters for the 0–1 m depth in the experimental field are shown in Table 5.

**Figure 8:**
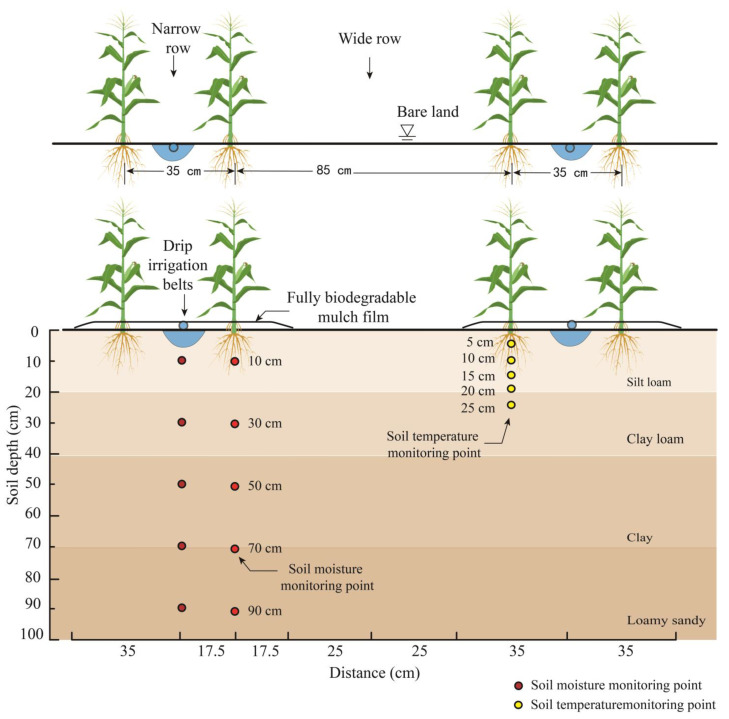
Planting patterns and soil moisture observation points.

The planting pattern followed “one film, one drip irrigation with two rows of corn” (Figure 8). The experiment included three induction periods, 60 days (BM60), 80 days (BM80), and 100 days (BM100), with a non-mulched treatment serving as a control (CK). White, transparent, fully biodegradable mulch with a width of 70 cm and thickness of 0.008 mm was provided by Shandong Tianzhuang Eco-Benign Plastics Technology Co., Ltd. (Jinan, Shandong Province, China). The spacing between adjacent drip irrigation tapes was 1.2 m, with a distance of 35 cm between the two rows (narrow rows), resulting in a planting density of 67,500 plants per hectare. The drip emitters were spaced 25 cm apart, with an irrigation rate of 1.8 L·h^−1^, and each plot was equipped with a rotary digital water meter to record the amount of water applied. The base nitrogen fertilizer (63 kg ha^−1^) was applied using integrated agricultural machinery. For the application of top-dressing nitrogen, urea was fully dissolved in a fertilizer tank to obtain an amide nitrogen solution, which was then applied under the mulch to the root zone via the water pressure difference. The top-dressing was added in three applications: 82.8 kg ha^−1^ at the jointing stage, 82.8 kg ha^−1^ at the tasseling stage, and 41.4 kg ha^−1^ during the grain-filling stage. The spring maize variety used in this study was Nonghua 106. Maize was planted on 29 April 2016, 27 April 2017, and 27 April 2018 and harvested on 26 September 2016, 24 September 2017, and 25 September 2018. The start and end dates of the key growth stages were recorded, and pest and weed management, along with agricultural machinery and agronomic practices, were conducted according to conventional local methods.

### 4.2. Data Measurement and Methods

#### 4.2.1. Meteorological Data

**Figure 9:**
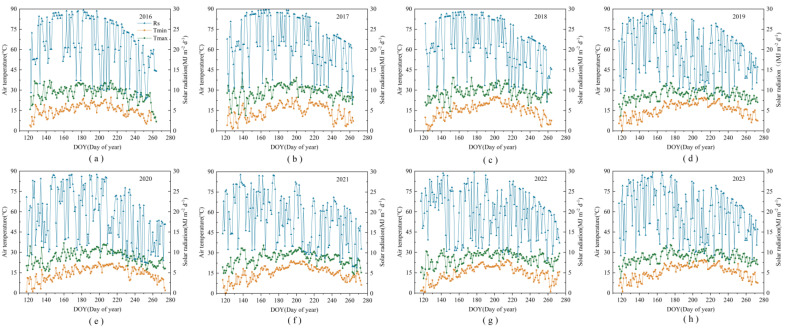
Maximum temperature (*T*_max_), minimum temperature (*T*_min_), and solar radiation (SR) during 2016, 2017, 2018, 2019, 2020, 2021, 2022, and 2023 (**a**–**h**).

Field climate data were collected using a weather station (HOBO U30 Onset; Onset Computer Corporation, Bourne, MA, USA) installed at the experimental site. Reference maize evapotranspiration (ET_0_) was calculated using the Penman–Monteith equation (Figure 9). Precipitation estimates recorded during the growing seasons from 2016 to 2023 were as follows: 272.03, 290.42, 212.56, 244.37, 262.99, 257.46, 252.56, and 414.89 mm. Notably, 2018 was classified as a low-flow year, whereas 2023 experienced above-average precipitation, making it a wet-flow year. The remaining years were classified as normal-flow years [26].

#### 4.2.2. Soil Water Content

The soil water content was monitored using an automatic soil water recorder (THL-TS-15; Beijing Kunlun Taiheng Technology Co., Ltd., Beijing, China.), and validation was conducted using the drying method. The automatic recorder collected data hourly, with monitoring points located directly beneath the drip irrigation tape and at depths of 10, 30, 50, 70, and 90 cm between the corn rows (Figure 8).

#### 4.2.3. Soil Temperature

The soil temperature was monitored using an automatic soil temperature recorder (THL-TWS-14; Beijing Kunlun Taiheng Technology Co., Ltd.). Soil temperature sensors were installed at depths of 5, 10, 15, 20, and 25 cm below the surface between the corn rows to continuously track changes in soil temperature in the plowed layer, with data recorded every hour.

#### 4.2.4. Fully Biodegradable Mulch Film Damage Ratio Statistical Method

After sowing, three observation points were randomly selected from the plots treated with fully biodegradable mulch. Observations were recorded every 10 days, and photos were taken 30 cm above the observation area. After geometric correction and background removal, the images were imported into CAD, and the damaged areas were outlined. As most of the damaged areas are circular or elliptical, the radius of each damaged area is statistically measured to simplify the assessment by considering half of the surface area of the sphere with the damaged hole [14]. The statistical formula for fully biodegradable mulch film damage ratio ($F_{d}$) is as follows:

(1) $$F_{d}=\frac{\sum_{i=1}^{n}A_{i}}{A_{d}}$$

(2) $$A_{i}=2\pi r^{2}$$

where $A_{d}$ is the total area of the region between the narrow rows of corn (cm^2^), $A_{i}$ is the surface area of the damaged biodegradable film within the region (cm^2^) for  i ranging from 1 to n, and r is the radius of the small hole (cm).

#### 4.2.5. Leaf Area Index, Yield, and WUE

For each plot, five representative plants with consistent growth were selected and marked with tags for continuous monitoring of the corn leaf area throughout the growth stages. The leaf area index (LAI) was calculated using the formula proposed by Watson [27].

(3) $$\mathrm{LAI}=S_{1}/S_{2}$$

where S_1_ is the measured leaf area (m^2^) and S_2_ is the corresponding ground area (m^2^).

At harvest, three 10 m long strips of double rows of corn were taken parallel to the drip irrigation tape from each plot. After harvesting all of the grains and allowing them to air-dry, the total weight was recorded and converted to yield per hectare. 

The measured groundwater depth in the experimental area was 8.5 m. Therefore, groundwater replenishment was neglected. Drip irrigation was used, and the land surface was leveled, allowing surface runoff to be neglected. The soil water balance equation and formula for calculating WUE were as follows:

(4) $$ET_{c}=I+P-\Delta W-D$$

(5) $$WUE=\frac{Y}{ET_{c}}$$

where *ET_c_* represents the total evapotranspiration (mm) and *I* and *P* denote the irrigation amount (mm) and effective rainfall (mm), respectively. ΔW is the change in water storage during the period used to calculate *ET_c_*, (mm). *D* refers to the deep drainage or rising capillary flow (mm). *WUE* stands for water use efficiency (kg mm^−1^ ha^−1^), and *Y* represents grain yield (kg ha^−1^).

### 4.3. Model Improvement

The DSSAT model includes modules for weather, soil, maize, and field management. In DSSAT, ET is typically estimated using the Priestley–Taylor model, which is based on solar radiation, rainfall, maximum temperature (*T*_max_), and minimum temperature (*T*_min_). Biodegradable film covers can increase the soil surface temperature and reduce soil evaporation during the early stages. However, this can also lead to a decrease in rainfall. As the film degrades, its insulating effect diminishes, resulting in increased soil evaporation, although some rainfall can still be utilized. The DSSAT model does not include a film cover module and assumes uniform irrigation across the entire field. In this study, we used drip irrigation, which is a form of localized irrigation. Therefore, the input values for irrigation, rainfall, and temperature were adjusted to improve the soil evaporation modulus.

#### 4.3.1. Irrigation Amount

The DSSAT model simulates soil moisture dynamics based on the assumption of uniform irrigation across the entire field. For instance, if the actual irrigation amount is 3 m^3^ ha^−1^, the DSSAT model assumes that the irrigation depth at any point in the field is 30 mm. In reality, the irrigation depth near the drip tape was greater than 30 mm. If we input a 30 mm irrigation depth into the model, it will underestimate the soil moisture near the maize roots under drip irrigation conditions. To improve the accuracy of the model, the irrigation data input was adjusted. Additionally, considering that plastic mulch is a significant influencing factor, we focused only on the soil moisture status in the area near the drip tape, specifically in the regions between the narrow rows of corn. The actual amount of irrigation, $Q_{real}$ (mm), was calculated using soil moisture data collected using automatic recording devices before and after irrigation using the following formula:

(6) $$Q_{real}=1000\times \left(\theta_{after}-\theta_{before}\right)\times H$$

In the equation, $\theta_{before}$ represents the soil moisture content before irrigation (cm^3^ cm^−3^), $\theta_{after\;}$ denotes the soil moisture content after irrigation (cm^3^ cm^−3^), and *H* is the depth of wet soil in the area between the narrow rows of corn (m). The actual irrigation amounts and model input values for 2016, 2017, and 2018 are listed in Table 6.

#### 4.3.2. Temperature

The warming effect also changes after the degradation of the biodegradable mulch. Therefore, the DSSAT model should account for the effect of soil temperature variation on maize growth following mulch degradation. Temperature compensation under biodegradable film coverage can be calculated using the following formula [28]:

(7) $$T_{max-f}=T_{max}+\Delta T_{a}$$

(8) $$T_{min-f}=T_{min}+\Delta T_{a}$$

(9) $$\Delta T_{a}=C\Delta T_{s}\frac{T_{a}-T_{b}}{T_{s}-T_{b}}$$

$T_{max-f}$ is the maximum air temperature after compensation under biodegradable film mulching and $T_{min-f}$ is minimum air temperature (°C), $\Delta T_{a}$ is the air temperature difference between biodegradable film mulching and no-film mulching (°C), C is the compensation coefficient, $\Delta T_{s}$ is the soil temperature difference between biodegradable film mulching and no-film mulching (°C), $T_{a}$ is the average air temperature, $T_{b}$ is the biological lower limit base point temperature of maize (8 °C), and $T_{s}$ is the daily soil temperature under no-film mulching (°C).

The compensation coefficient (C) was determined using the following equation [15]:

(10) $$C=\frac{T_{cum-a-s}-T_{cum-a-f}}{T_{cum-s-f}-T_{cum-s-s}}$$

(11) $$T_{cum}=\sum_{i=1}^{n}(T_{ai}-T_{b})$$

where $T_{cum-a-s}$ is the effective air accumulated temperature under no-film mulching (°C), $T_{cum-a-f}$ is the effective air accumulated temperature under biodegradable film mulching (°C), $T_{cum-s-f}$ is the soil accumulated temperature under no-film mulching (°C), $T_{cum-s-s}$ is the effective soil accumulated temperature under biodegradable film mulching (°C), and $T_{ai}$ is the average air temperature on day *i*.

#### 4.3.3. Soil Evaporation

In the DSSAT model, *E_s_* is divided into two stages: stage I (constant-rate stage) and stage II (falling-rate stage). In stage I, *E_s_* was not affected by the variation in SWC, and *E_s_* in this stage was computed using the following equation:

(12) $$E_{si}=\sum E_{s0}$$

where *E_si_* is the cumulative soil evaporation (mm d^−1^) and *E_s_*_0_ is the actual soil evaporation on day i.

In stage II, *E_s_* depended on the water vapor flux through the shallow soil layer, which was calculated using the following equation:

(13) $$E_{si}=\alpha \sqrt{t}$$

where α is the soil hydraulic characteristic parameter (mm d^−0.5^) and t is the evaporation time (d).

The actual soil evaporation was related to the potential soil evaporation, and the potential soil evaporation was calculated from the potential *ET*, calculated using the default Priestley–Taylor formula of the DSSAT model [29]. The potential evapotranspiration (*ET_p_*) and (*ET_c_*) under the biodegradable film mulching were obtained using the following equations:

(14) $$ET_{p}=ET_{c}\times 1.1$$

(15) $$ET_{c}=SLANG\times \left(2.04\times 10^{-4}-1.83\times 10^{-4}\times ALBEDO\right)\times \left(TD+29\right)$$

(16) $$TD=0.6\times T_{max-f}+0.4T_{min-f}$$

where *ET_p_* is the potential evapotranspiration rate (mm/d); *ET_c_* is the equilibrium evaporation rate, mm/d; *SLANG* is the solar radiation (MJ m^−2^ d^−1^); *ALBEDO* is the reflectance of the soil–maize surface, set to 0.23 in DSSAT 4.6; and *TD* is the approximation of average daily temperature (°C).

Because the DSSAT model does not account for the impact of biodegradable film coverage on soil evaporation, a small amount of soil moisture evaporates from the damaged areas of the corn-planting holes before the film fully degrades. As degradation progressed, the soil evaporation rate was positively correlated with the rate of film damage. Therefore, the improved evaporation rate *E_s__i_* was determined using the following equation:

(17) $$E_{si}=E_{s}\times F_{d}$$

#### 4.3.4. Precipitation

Before the degradation of the fully biodegradable film, rainfall in the area near the drip tape could be neglected because of the obstruction caused by the film. After degradation, the utilization of rainwater is related to the damage rate of the film. The model input for precipitation $P_{e}$ was calculated using the formula described by Feng et al. [23].

(18) $$P_{e}=P\times \lambda$$

(19) $$\lambda =54.7167-2.258P+1.4297F_{d}+0.0310P^{2}-0.0238F_{d}^{2}+0.045P\cdot F_{d}$$

In this context, $P_{e}$ represents the amount of rainfall accumulation in the area under the film (mm), P denotes the total amount of subsequent rainfall (mm), λ is the effective infiltration rate of the rainfall, and $F_{d}$ is the film damage ratio (%).

### 4.4. Model Evaluation

The prediction performance of the model was quantitatively evaluated based on the mean relative error (*MRE*, %), normalized root mean square error (*nRMSE*, %), and coefficient of determination (*R*^2^).

(20) $$\mathrm{MRE}=\frac{1}{n}\sum_{i=1}^{n}\frac{\left|S_{i}-O_{i}\right|}{S_{i}}\times 100\%$$

(21) $$nRMSE=\frac{\sqrt{\frac{1}{n}\sum_{i=1}^{n}{\left(S_{i}-O_{i}\right)}^{2}}}{O_{max}-O_{min}}\times 100\%\;$$

(22) $$R^{2}={\left[\frac{\sum_{i=1}^{n}\left(S_{i}-\bar{S}\right)\left(O_{i}-\bar{O}\right)}{\sqrt{\sum_{i=1}^{n}{\left(S_{i}-\bar{S}\right)}^{2}}\sqrt{\sum_{i=1}^{n}{\left(O_{i}-\bar{O}\right)}^{2}}}\right]}^{2}$$

where *S_i_* and *O_i_* represent the simulated and measured values, respectively, and *n* represents the number of data points.

## 5. Conclusions

To accurately simulate maize growth under fully biodegradable mulch, this study utilized the evapotranspiration redistribution characteristics associated with such mulch. Moreover, the soil evaporation module was improved, the irrigation and rainfall input values were corrected using the one-dimensional water balance principle, and the effective air temperature compensation coefficient was calculated based on the warming effects of the mulch. The model was also optimized for genetic parameters of spring corn using field test data. This calibrated and verified DSSAT model effectively simulates the phenological period, leaf area index (LAI), yield, and soil moisture content of spring corn covered with biodegradable mulch across different coverage periods. Its simulation accuracy is characterized by an MRE < 10%, an nRMSE < 12%, and an *R*^2^ ≥ 0.80. This study offers enhanced methods for applying the DSSAT model to simulate maize under fully biodegradable mulch in a drip irrigation system.

In addition, based on experiments conducted from 2016 to 2018 and model predictions of yield and water use efficiency from 2019 to 2023, this study recommends that the Xiliaohe Plain and similar climatic areas should adopt fully biodegradable mulch with an induction period of approximately 80 days to ensure high yields across different hydrological years. The results of this study provide a scientific foundation and technical support for predicting the productivity of maize covered with fully biodegradable mulch and for optimizing the management of agricultural water and heat resources in the semi-arid regions of Northeast China in the context of climate change. In future research, continuous field experiments will be conducted in arid and semi-arid regions of China (across different temperature zones), considering the impacts of water stress and extreme weather on maize to further enhance the DSSAT model.

## Figures and Tables

**Table 1:** Compensatory coefficient for the cumulative air temperature under fully biodegradable film mulching.

Year	Treatments	Temperature Increase Compensation Coefficient		
		Sowing to Emergence	Emergence to Elongation	Elongation to Tasseling
BM60	2019	1.47	0.35	0.10
	2020	1.38	0.32	0.12
	2021	1.59	0.43	0.13
	2022	1.61	0.37	0.09
	2023	1.42	0.33	0.13
	Mean	1.49	0.36	0.11
	CV	5.58%	9.89%	13.01%
BM80	2019	1.48	0.41	0.15
	2020	1.38	0.38	0.13
	2021	1.59	0.45	0.16
	2022	1.61	0.39	0.11
	2023	1.42	0.36	0.15
	Mean	1.50	0.40	0.14
	CV	5.55%	7.02%	11.66%
BM100	2019	1.49	0.44	0.21
	2020	1.39	0.40	0.16
	2021	1.59	0.47	0.20
	2022	1.62	0.42	0.16
	2023	1.42	0.39	0.18
	Mean	1.50	0.42	0.18
	CV	5.51%	6.18%	10.23%

**Table 2:** Genetic parameters for Nonghua 106 summer maize.

Variety	P1	P2	P5	PHINT	G2	G3
Nonghua 106	196	0.55	858	45	980	6.2

**Table 3:** Simulation results for the phenological period, yield, and ET_C_ based on the modified DSSAT model for 2016, 2017, and 2018.

Treatment		Emergence			Tasseling			Filling			Harvest			Yield (kg/ha)			ETc (mm)		
		Sim.	Obs.	MRE (%)	Sim.	Obs.	MRE (%)	Sim.	Obs.	MRE (%)	Sim.	Obs.	MRE (%)	Sim.	Obs.	MRE (%)	Sim.	Obs.	MRE (%)
2016	BM60	135	136	0.74%	203	205	0.99%	228	229	0.44%	262	264	0.76%	13,301	13,484	1.37%	443.25	450.69	1.68%
	BM80	135	136	0.74%	202	205	1.49%	227	229	0.88%	261	264	1.15%	13,015	12,995	0.15%	438.22	445.52	1.67%
	BM100	135	136	0.74%	201	205	1.99%	226	228	0.88%	260	264	1.54%	12,409	12,471	0.50%	427.07	435.39	1.95%
2017	BM60	136	138	1.47%	206	206	0.00%	231	233	0.87%	265	265	0.00%	12,890	13,379	3.79%	491.74	471.35	4.15%
	BM80	136	138	1.47%	205	206	0.49%	230	233	1.30%	264	265	0.38%	12,361	12,848	3.94%	490.99	470.43	4.19%
	BM100	136	138	1.47%	204	206	0.98%	229	233	1.75%	263	265	0.76%	12,351	12,727	3.05%	484.87	464.97	4.10%
2018	BM60	135	134	0.74%	206	204	0.97%	231	230	0.43%	265	263	0.75%	11,305	11,193	0.99%	415.44	435.95	4.94%
	BM80	135	134	0.74%	206	204	0.97%	231	230	0.43%	265	263	0.75%	12,125	12,461	2.77%	411.36	431.62	4.93%
	BM100	135	134	0.74%	205	204	0.49%	230	228	0.87%	264	263	0.38%	12,330	12,732	3.26%	402.26	423.19	5.20%

**Table 4:** Prediction results for rainfall, yield, and water use efficiency for different induction periods under fully biodegradable film mulching treatments.

Treatment		Precipitation	ETc	Yield	WUE
		(mm)	(mm)	(kg ha^−1)^	(kg mm^−1^ ha^−1^)
2019	BM60	192.53	449.74	12,659.15	28.15
	BM80	177.81	434.68	12,471.10	28.69
	BM100	168.97	423.74	12,223.73	28.85
2020	BM60	203.98	459.85	13,692.62	29.78
	BM80	203.98	455.12	13,531.60	29.73
	BM100	184.86	439.28	13,114.28	29.85
2021	BM60	242.34	449.90	12,903.12	28.68
	BM80	226.56	435.48	12,715.48	29.20
	BM100	204.58	412.70	12,140.05	29.42
2022	BM60	209.05	457.10	12,316.49	26.94
	BM80	192.87	442.48	12,284.17	27.76
	BM100	183.42	434.80	12,179.04	28.01
2023	BM60	382.13	498.03	11,819.63	23.73
	BM80	364.80	480.30	11,442.29	23.82
	BM100	352.64	467.84	11,163.77	23.86

**Table 5:** Soil texture and hydraulic parameters for a depth of 0–1 m at the test site.

Soil Layers	Soil Types (%)			Field Capacity	Saturated Water Content	Wilting Point	Bulk Density
(cm)	Clay	Silt	Sand	(cm^3^ cm^−3^)	(cm^3^ cm^−3^)	(cm^3^ cm^−3^)	(g cm^−3^)
0–20	10.54	52.7	36.76	0.26	0.40	0.08	1.39
20–40	29.54	48.81	21.65	0.34	0.45	0.08	1.38
40–70	40.67	39.15	20.18	0.45	0.53	0.10	1.41
70–100	1.41	25.58	73.01	0.17	0.38	0.05	1.52

**Table 6:** Actual value (Act) and input model value (Inp) of the irrigation depth in 2016, 2017, and 2018.

Year	Irrigation Depth (mm)								
2016	Date	9 May	2 June	9 July	22 July	2 August	8 August	14 August	25 August
	Act	30	30	30	15	10	20	20	20
	Inp	51	53	44	27	31	42	46	39
2017	Date	8 May	11 June	27 June	2 July	17 July	22 July	2 August	
	Act	30	30	30	30	20	10	10	
	Inp	52	56	50	46	39	18	24	
2018	Date	9 May	18 May	10 June	22 June	17 July	1 August	9 August	
	Act	15	30	30	30	30	30	30	
	Inp	33	59	58	52	42	37	40	

## Data Availability

Data will be made available on request.
